# What is the cost of equity in assistive technology? A tool for calculating assistive products and services

**DOI:** 10.3389/fresc.2026.1802496

**Published:** 2026-07-16

**Authors:** Natasha Layton, Em Bould, Libby Callaway, Emma Friesen Osborne, Natasha Brusco

**Affiliations:** 1 Rehabilitation, Ageing and Independent Living (RAIL) Research Centre, School of Primary and Allied Health Care, Faculty of Medicine, Nursing and Health Sciences, Monash University, Victoria, Australia; 2 Department of Occupational Therapy, School of Primary and Allied Health Care, Faculty of Medicine, Nursing and Health Sciences, Monash University, Melbourne, Australia; 3 Medical School – Rural Clinical School Research Centre, The University of Queensland, Queensland, Australia

**Keywords:** assistive technology, equity, Indigenous, rurality, service provision

## Abstract

This paper aims to address a technical conundrum in accurately costing and delivering assistive products and services in the context of market-shaping strategies designed to promote assistive technology (AT) access. Developed in response to Australian Government policy changes regarding provision of AT to community-dwelling older adults, the *AT SERVE (Assistive Technology—Services, Equity, Resources, Valuation, and Enablement)* Tool is introduced. This novel interdisciplinary approach informs equitable modelling of AT costs by incorporating both assistive products and essential services that enable the selection, fitting, maintenance, and outcome measurement vital for safe and effective use. The methodology utilised interdisciplinary systems thinking to bring together targeted, policy-focussed reviews of international and Australian statutes and practice guidelines. Key components included (1) assistive product classification; (2) delineation of service provision elements; (3) workforce cadres required for service delivery; (4) healthcare intervention guidance regarding rurality, indigeneity, assistive product complexity, and risk; and (5) applied health economics cost modelling appropriate for a government context. The exemplar presented relates to the Australian context but can be adapted to the intersectional factors relevant in any setting.

## Introduction

Assistive technology (AT) optimises functioning and reduces the experience of disability for people living with functional limitations (1, 2). Such limitations may arise due to the presence of disease, disability, and/or the functional sequelae of ageing. The burden of disease associated with these functioning limitations is substantial and continues to grow. Universal access to assistive products has been recognised as a critical link to the realisation of the World Health Organization's Sustainable Development Goal of leaving no one behind (3). However, there is a gap in provision of AT to those who need it the most. This concern has been highlighted in a number of global directives, including a World Health Assembly resolution on improving access to AT (4) and a subsequent Global Report on AT (1). Recommendations stemming from these directives include enacting universal healthcare through the building of priority assistive product lists and coupling the provision of assistive products with services that enable safe and effective use, including selection, fitting, maintenance, and outcome measurement (5).

A wave of new thinking is evident in the AT ecosystem (6), including the call for a “new economics” for AT that is mission-led, epistemically just, and ecosystem-focussed. Multiple market-shaping initiatives are being developed and tested in various contexts (7, 8). This growing focus on market-shaping strategies to promote access to assistive products, combined with calls for research into the determinants of equitable AT access across national, regional, and global levels, underscores the timeliness of this perspective article.

## Aims

This perspective article aims to present an approach to equitable modelling of AT costs, inclusive of both assistive products and services.

## Background and context

Governments must allocate resources as part of the social contract (9). Priority setting in healthcare and public health is a key mechanism for resource allocation. Priority setting can lead to social justice concerns. Economic evaluation has a role to play in addressing social justice concerns, that is actioning the “moral imperative to avoid and remediate unfair distributions of societal disadvantage” [(10), p. 27].

Research in the AT field has generally focussed on the pricing of assistive products themselves, often neglecting AT service delivery costs, the steps involved, and the workforce cadres required (11). However, as Tay-Teo et al. stated, “From a technical aspect, decision-makers and system managers should ensure the allocative efficiency of the overall system. In its broadest sense, this means what services and products should be included in the package to maximise welfare” [(12), p. 5110]. This suggests that resource allocation must include methods to accurately specify the cost of both assistive products and the services necessary for their deployment.

The most substantive investment in service delivery research occurred through Europe's TIDE and HEART studies, which generated a body of knowledge adopted by civil society (13–15). Building on this work, Andrich et al. (16) developed a robust methodology over several decades to capture the costs and outcomes of AT provision. The authors achieved this by utilising “cost” as an economic concept describing the use of resources. As such, they were able to convert non-fiscal resource use into measurable units via a “valuation” process (16), arriving at a social cost inventory analysis instrument applicable to workforce cadres involved in AT service provision (17, 18). In another example, a methodology has been developed for cost–benefit analysis of AT for application to certain diagnoses like dementia (19, 20). Such forecasting has, to varying degrees, attempted to make explicit “invisible” workforce and service steps, without which an assistive product may be poorly fitted, inappropriate for the environment or task, abandoned prior to or following actual use, or cause functioning difficulties.

This exemplar integrates the body of work on specifying products and services, with the notion of cost-based equity weights as one practical mechanism to enact equity (21). Cost-based equity weighting reduces the cost of more expensive, equity-enhancing, population-specific interventions by applying a weight proportional to the cost differences between population-specific delivery and mainstream delivery (22). Equity weights for use in the economic evaluation of primary healthcare interventions have been developed (23) and widely cited as a method to achieve health equity (24–26). They have been applied to indigenous populations, including in New Zealand (27), and in international contexts, including South Africa (28) and Europe (29)*.*

## Quantifying “equity”

The “mixed economy” of AT product and service providers is complex. AT provision is influenced by location (sometimes down to locality or region, as well as country or economic zone), diagnosis, eligibility for schemes, and availability of schemes (30, 31). Nonetheless, global principles provide a solid foundation. These include the notion that health equity measures are required to redress health inequities and that these equity measures must take into account social determinants of health (32). Internationally agreed assistive product interventions (33), common quality service provision principles (34), and international standards (35) provide an empirical basis from which to develop a formula within which contextual differences can be captured (names, costs) and worked through.

In Australia, AT access has been historically limited, inequitable, and complex in nature (36). Over the past decade, Australian AT researchers have examined AT policy and resource allocation practices (37), as well as priority setting in disability and aged care (38, 39). Economic models which take into account AT equity and access have also been developed, introducing the term “wraparound supports” as a descriptor of the workforce cadres central to service steps necessary for safe and effective AT provision (40, 41).

The economic model presented in this perspective article was developed in 2024–2025 in response to Australian Government policy changes in the delivery of AT to community-dwelling older adults in an effort to inform AT programme resource allocation in Australia.

## Method

### Step 1 Reflecting on positionality

Our multidisciplinary research team members came from diverse professional backgrounds, including occupational therapy, physiotherapy, rehabilitation engineering, and psychology, with research expertise in data science, health policy research, health economics, and qualitative inclusive research. In addition, the team members had a combined 50 years of clinical practice experience in AT assessment and service provision.

Reflexivity steps included the following: (1) bracketing profession-specific assumptions regarding the scope of practice, as guided by Australian statutory regulation (AHPRA) (55) and self-regulation bodies (NASRHP) (56); (2) research team meetings supported by field notes to iteratively contextualise the evidence collated during Step 2, aligning with the specific Australian government context and the commissioned work; and (3) applying interdisciplinary systems thinking to develop nuanced and context-specific health equity considerations with which to build a cost-based equity-weighting calculation tool (3, 42).

### Step 2 Development of the AT SERVE tool

Tool development should be informed by evidence and refined through consultation. For the Australian context, the following steps were undertaken, noting substantive prior academic and clinical work, partly commissioned by government, which informed the set of evidence drawn together:

- selection of assistive product classification using AS/NZS ISO 9999, the Australian adoption of the international standard for the classification of assistive products (35);
- delineation of AT service provision elements based on a recent scoping review of practice guidelines undertaken by the authors (43);
- identification of the workforce cadres required for service delivery, based on guidance from government as well as prior work by the authors (44);
- targeted policy review of relevant international statutes (45), Australian legislation and programme guidance (46), Australian healthcare intervention guidance addressing equity, rurality, and indigeneity (22, 47, 48), and government and engineering approaches to quantifying assistive product risk (49–51);
- selection of applied health economics cost modelling from prior commissioned government work by the authors (40, 41).

### Step 3 Consultation and preliminary validation

The combined evidence was drafted into a cost-based equity-weighting calculation tool, built in Microsoft Excel and termed the *AT SERVE (Assistive Technology—Services, Equity, Resources, Valuation, and Enablement)* Tool. The draft version of *AT SERVE* was presented in written and video conference formats to both government (the department commissioning the work) and the AT sector in Australia^1^ (52) for peer review and feedback. These reviews endorsed the components of the tool, which was then further edited for clarity and finalised as a fillable Microsoft Excel spreadsheet.

## Results

**Figure 1:**
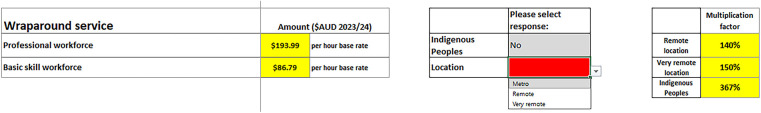
Equity-weighting approach of the AT SERVE (Assistive Technology - Services, Equity, Resources, Valuation, and Enablement) tool.

**Figure 2:**
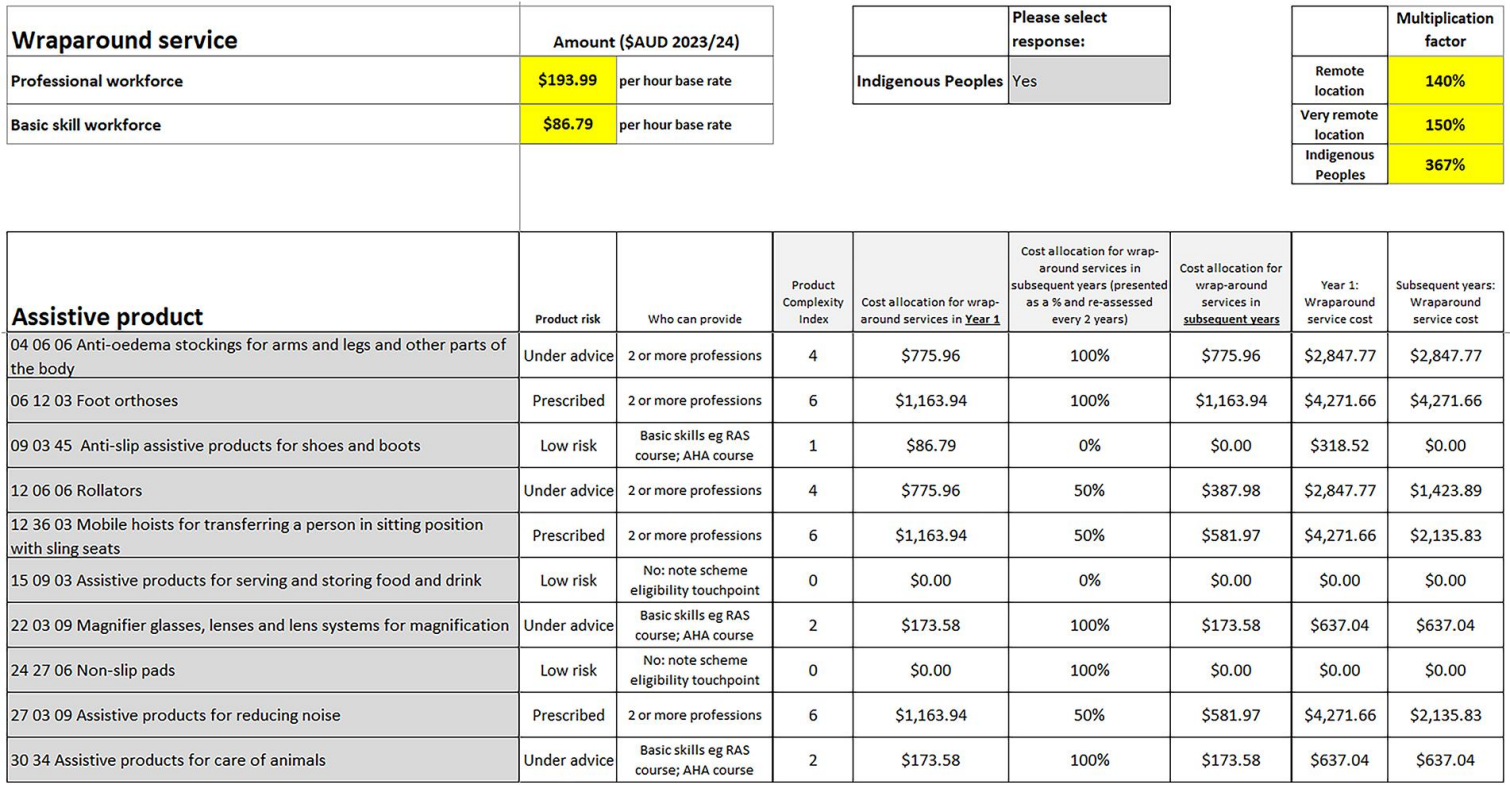
Worked *AT SERVE* example applied to the Australian aged care context.

The *AT SERVE* Tool adopts a cost-based equity-weighting approach that recognises and systematically incorporates contextual factors such as place (rurality and remoteness) (47, 48) and indigeneity (22). As illustrated in Figure 1, wraparound services for assessment and safe recommendation of AT (provided by appropriately qualified and skilled assessor workforces) are estimated at an hourly rate. Multiplication factors are then applied based on the dimensions of rurality (remote or very remote in the Australian context) or indigeneity. There are separate dropdowns to select either the indigenous filter or location; however, as shown in Figure 2, when Indigenous Peoples are selected, remote/very remote locations cannot also be selected.

*AT SERVE* is not intended to replace clinical judgement or assessment. Rather, it is an adjunct formula that can be applied to determine costs for assistive products and workforce cadres.

Supporting the formula is a database of assistive products and a set of assessor workforce type and cost variables. Figure 2 presents the data items generated for the Australian context, applicable to many other settings. These include the following: (1) assistive product; (2) product risk level; (3) assessor qualifications required; (4) Product Complexity Index (PCI), calculated depending on the need for wraparound services and the requisite assessor workforce; (5) ownership and repair responsibility; and (6) review or follow-up expectations for wraparound services in subsequent years.

To illustrate, a worked example is provided in Figure 2. Column 1 catalogues assistive products according to the ISO 9999 classification and terminology standard (35). The rows depict sample assistive products from each ISO 9999 class. Class 28 (Assistive products for work activities and participation in employment) is excluded, as Australian aged care funding for AT does not cover assistive products for employment (49).

Column 2 denotes a product's risk classification across three categories: low risk, under advice, or prescribed. This product risk classification is used in Australian aged care policy (49) and disability policy (51), and is recommended by Engineers Australia (50), but may differ in other contexts.

Column 3 identifies minimum qualifications and competencies of the assessor workforce providing wraparound supports. In this Australian example, options include “basic skills,” which are minimum assessor qualifications within a regional assessment service (RAS) or aged care workforce, such as allied health assistants (AHA) with certificate or diploma training. Other jurisdictions will have different workforce cadres, scopes of practice, and costings.

Column 4 contains the PCI, which translates into minimum assessor hours needed to provide wraparound services. The PCI is calculated by multiplying the product risk classification (Column 2 “Product risk”; *Low risk* = 1, *Under advice* = 2, *Prescribed* = 3) with assessor competencies [Column 3 “Who can provide”; *None* = 0; *Basic skills* = 1, *Professional (2 or more professions/Specific qualification required)* = 2]. Possible results are 0, 1, 2, 4, or 6.

The remaining columns outline the costs for one or multiple years. This functionality recognises that some products—for example, an anti-oedema stocking (shown in Figure 2)—will not be suitable for multi-year use, refurbishment, or maintenance, so costs are allocated in the first year. Other products, such as mobile hoists, require AT service provision steps such as service maintenance or an annual review (34), so costs can be allocated across subsequent years. Worked examples in Column 2 include low risk (row 3, 6, 8), under advice (row 1, 4, 7, 10), and prescribed (row 2, 5, 9), thereby demonstrating the tool's sensitivity to different product tiers.

## Discussion

Accurate costing of both assistive products and services within the AT ecosystem is essential to calculate, invest in, and assure effective provision of AT such that the return on investment is realised. Cost-based weights have been identified as a pragmatic method of equity weight construction, which is both understandable to policymakers and sensitive to the needs of target groups (22). The *AT SERVE* Tool draws on international statutes and practice guidelines, published healthcare intervention guidance (including rurality and indigeneity), health economics, and interdisciplinary systems thinking to inform equitable modelling of AT costs inclusive of both assistive products and services.

Internationally, there have been calls for market-shaping interventions that include reducing transaction costs, increasing market information, and balancing supplier and buyer risks (8). The *AT SERVE* Tool offers a transparent economic modelling framework that allows for pricing of both product and service weightings—an important part of such interventions, particularly in countries were intersectionality can influence AT equity. Provision of assistive products has traditionally been conducted through local providers, manufacturing products within a limited price range (42). When considering market-shaping strategies to promote access to assistive products, recognised practice steps—including the payment for and provision of assistive services to select, fit, and follow up assistive product use—must not be overlooked, and the cost of these services must be factored into AT programmes (52, 53). Historically, however, whilst AT comprises both assistive products and the services to provide those products safely and effectively (54), the evidence base lacks implementation tools capable if integrating products and services and accurately costing and measuring them (1). Whilst the *AT SERVE (Assistive Technology—Services, Equity, Resources, Valuation, and Enablement)* Tool was developed to inform equitable costing of AT for older adults in Australia, its variables and related metrics can be adapted to different contexts, making it globally applicable.

## Limitations

The *AT SERVE* Tool has several limitations. First, it was developed for and applied as an exemplar in a single AT programme in Australia, with costings, workforce assumptions, and programme boundaries reflecting that setting. This developmental work was part of commissioned outputs for government and as such used highly pragmatic synthesis of evidence and targeted expert consultations to produce a cost-based equity-weighting calculation for testing. In other contexts, more standard review methodologies may apply (e.g., systematic, narrative, or scoping reviews of core concepts for particular settings) and/or systematic consultation methods may be appropriate (e.g., Delphi method, structured workshops). Second, the tool focuses on cost identification and equity-informed weighting, rather than outcome measurement, and therefore does not, on its own, permit statements about cost effectiveness or value for money in comparative terms. Future research should therefore examine the application of the *AT SERVE* Tool in other national and regional contexts, both in Australia and internationally. In addition, research could explore the tool’s integration with formal economic evaluation methods and test its utility for informing policy decisions and funding equity.

## Conclusion

Whilst there is potential for market-building and -shaping strategies to transform the global landscape of assistive product provision, to date, much of the focus has been on the costing of assistive products. Less attention has been given to the weighting of factors that influence product supply, including costing of assistive services that are a vital part of the evidenced good practice in AT provision. This perspective paper has offered an economic model exemplar—the *AT SERVE (Assistive Technology—Services, Equity, Resources, Valuation, and Enablement)* Tool—which recognises the AT ecosystem required to achieve effective and efficient outcomes. *AT SERVE* was developed in response to some of the assistive technology reforms underway in Australia. Whilst the exemplar provided relates to that regional context, further work is warranted to test the methodological approach and its application in systems, services, and policies both nationally and internationally.

## Data Availability

Publicly available datasets were analysed in this study. These data can be found here: https://www.iso.org/standard/72464.html. The *AT SERVE* tool is available upon reasonable request from the corresponding author.

## Footnotes

^1^ https://www.arata.org.au/access-&-funding/towards-a-national-assistive-technology-alliance/
